# One-step RT-qPCR assay for ZIKV RNA detection in *Aedes aegypti* samples: a protocol to study infection and gene expression during ZIKV infection

**DOI:** 10.1186/s13071-020-4002-x

**Published:** 2020-03-14

**Authors:** Ricardo Vieira Araujo, Fabiana Feitosa-Suntheimer, Alexander S. Gold, Berlin Londono-Renteria, Tonya M. Colpitts

**Affiliations:** 1 Department of Microbiology, Boston University School of Medicine, Boston, MA USA; 2 National Emerging Infectious Diseases Laboratories, Boston University, Boston, MA USA; 3 Climate Division, Ministry of Science, Technology, Innovations and Communications, Brasilia, DF Brazil; 4 Department of Entomology, Kansas State University, Manhattan, KS USA

**Keywords:** *Aedes aegypti*, Zika virus, One-step qRT-PCR, Mosquito gene expression

## Abstract

**Background:**

Zika virus (ZIKV) is transmitted to humans during the bite of an infected mosquito. In a scenario of globalization and climate change, the frequency of outbreaks has and will increase in areas with competent vectors, revealing a need for continuous improvement of ZIKV detection tools in vector populations. A simple, rapid and sensitive assay for viral detection is quantitative reverse transcription polymerase chain reaction (qRT-PCR), yet oligos optimized for ZIKV detection in mammalian cells and samples have repeatedly shown high background when used on mosquito ribonucleic acid (RNA). In this paper, we present a one-step qRT-PCR protocol that allows for the detection of ZIKV in mosquitoes and for the evaluation of gene expression from the same mosquito sample and RNA. This assay is a less expensive qRT-PCR approach than that most frequently used in the literature and has a much lower background, allowing confident detection.

**Methods:**

Our new oligo design to detect ZIKV RNA included *in silico* analysis of both viral and mosquito (*Ae. aegypti* and *Ae. albopictus*) genomes, targeting sequences conserved between Asian and African ZIKV lineages, but not matching *Aedes* genomes. This assay will allow researchers to avoid nonspecific amplification in insect samples due to viral integration into the mosquito genome, a phenomenon known to happen in wild and colonized populations of mosquitoes. Standard curves constructed with *in vitro* transcribed ZIKV RNA were used to optimize the sensitivity, efficiency and reproducibility of the assay.

**Results:**

Finally, the assay was used with success to detect both ZIKV RNA in infected mosquitoes and to detect expression of the Defensin A gene, an antimicrobial peptide (AMP) involved in *Aedes aegypti* immune response to virus infection.

**Conclusions:**

The experimental approach to detect ZIKV RNA in *Aedes aegypti* presented here has demonstrated to be specific, sensitive and reliable, and additionally it allows for the analysis of mosquito gene expression during ZIKV infection.
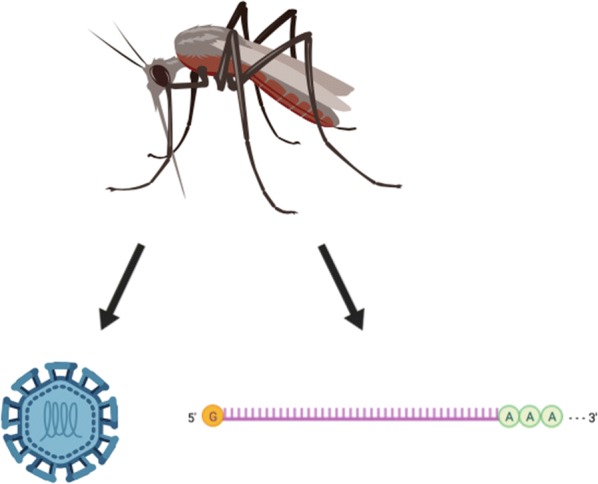

## Background

Zika virus (ZIKV) was first isolated in Uganda, from a sentinel rhesus macaque in 1947 [1]. It is an arthropod-borne virus (arbovirus) belonging to the genus *Flavivirus* of the *Flaviviridae* family. ZIKV infection in humans usually results in mild disease or asymptomatic infections; however, it can develop into to severe symptoms that can be lethal. The symptomatology can include fever, rash, arthritis and/or arthralgia and/or myalgia, conjunctivitis and fatigue. Neurological complications caused by ZIKV infection were reported in adults (Guillain–Barré syndrome) and neonates (congenital malformations including microcephaly) [2].

Before a major outbreak of Zika cases in 2007 at the Pacific Island of Yap in the Federate States of Micronesia [3], ZIKV infections occurred in Africa and Asia without much attention. In 2015, the first Zika cases were reported in the Americas (Brazil) and quickly spread to more than 20 countries throughout the Caribbean, South, Central, and North Americas [4–7]. In 2016–2017, Zika fever autochthonous cases were reported in USA, in the states of Texas and Florida [8, 9].

ZIKV is transmitted to humans by the bite of an infected mosquito. The main vectors associated with transmission in the urban cycle are *Aedes aegypti* and *Aedes albopictus*. In the current state of globalization and climate change, the frequency of human disease outbreaks related to arboviruses, including Zika, has increased in urban centers with competent vectors [10–13], revealing a need for continuous improvement of ZIKV detection in vector populations. In addition, there has been increased research involving ZIKV infection in the mosquito as an improved understanding of both pathogenesis and interactions in the vector will be crucial information. As such, successful detection of ZIKV infection in mosquito cells and samples is an important component of laboratory work involving the virus and vector.

Molecular detection of ZIKV ribonucleic acid (RNA) in mosquitoes can be challenging due to the limited number of primers and probes published [14–27], as well as the presence of viral genetic material integrated in mosquito genomes [28, 29], which can reduce the specificity for RNA detections by quantitative reverse transcription polymerase chain reaction (RT-qPCR) [30]. Most of these ZIKV oligos were optimized in mammalian cells and samples and show often-unresolvable background when used to detect infection in mosquito tissues.

In recent studies utilizing RT-qPCR to detect ZIKV RNA in mosquitoes, which mostly analyzed vector competence, it is possible to observe a methodological trend favoring the utilization of hydrolysis probes as a fluorescent label (83% of the papers in literature) and the use of viral RNA extraction kits to obtain the RNA templates (56% of the studies). However, the number of studies could actually be higher since some of them do not specify the extraction RNA method used for the experiments [9, 14, 16, 18–23, 31–51]. Although this approach has demonstrated to be relatively effective in detecting ZIKV RNA, since the isolation of viral RNA is prioritized, it does not permit the study of the gene expression in mosquito genes during viral infection using the same samples. The study of mosquito gene expression during ZIKV infection could elucidate phenomena not fully understand regarding ZIKV and mosquito interactions, as vector competence varies in mosquito populations infected with ZIKV isolates from different geographical regions [52–56]. In addition, these are very expensive approaches, a potential barrier to vector surveillance in developing countries.

In this study, we present a one-step qRT-PCR protocol that both detects ZIKV RNA and can be used to evaluate gene expression from the same sample of infected *Ae. aegypti*. In order to avoid nonspecific ZIKV RNA detection due to possible viral integration in the mosquito genome, *in silico* analysis of the *Ae. aegypti*, *Ae. albopictus* and ZIKV genomes were conducted to find sequences conserved between Asian and African ZIKV phylogenetic lineages [57, 58] but divergent from *Aedes* spp. genomes. Primers were designed to detect ZIKV RNA using these determined target regions. Primers were tested on *in vitro* transcribed ZIKV RNA as well as RNA samples from mosquitoes infected with ZIKV and the positive mosquito samples were used for transcriptional level analysis of Defensin A, an antimicrobial peptide (AMP) involved in *Ae. aegypti* immune response [59, 60].

## Methods

### Cell culture and virus growth

The Vero (ATCC CCL-81) cell line was used for growing ZIKV Puerto Rico-PRVABC59 (a kind gift of Dr Stephen Higgs, KSU, USA) and MR766 (BEI Resources, NR-50065) strains. Cells were grown at 37 °C and 5% CO_2_ in Dulbecco’s modified Eagle’s medium (DMEM) with 10% heat-inactivated fetal bovine serum (Gemini Bio, California, USA) and 1% penicillin-streptomycin (Gibco, Thermo Fisher Scientific, Massachusetts, USA). Infected cells were propagated for 5–7 days before supernatant collection and/or RNA extraction.

### Mosquitoes rearing and infection

*Aedes aegypti* (Rockefeller strain) were used in all experiments. Mosquito colony was maintained in a secure insectary (arthropod containment level 3, ACL3). The mosquitoes were bred and maintained in a controlled atmosphere (27 °C, 80% relative humidity and a 12 h light/dark cycle). Larvae were fed with powdered fish food (Tetra, Spectrum, Wisconsin, USA) and adult mosquitoes had access to 10% sucrose solution *ad libitum*. Female mosquitoes (7–14 days-old) were infected by blood-feeding using a Hemotek with ZIKV infected cell supernatant mixed with serum-inactivated human blood from healthy donors (ZenBio, North Carolina, USA), in a 1:1 proportion. The final titer for ZIKV PRVABC59 strain, in the blood solution, was 4 × 10^6^ plaque-forming unit (PFU)/ml. Mosquitoes were allowed to blood feed for 30 min, then ice-anesthetized and non-engorged females were removed. At 7 days post-infection, the fed mosquito whole bodies were homogenized in lysis buffer (RLT buffer, Qiagen, Maryland, USA) supplemented with β-mercaptoethanol (10 µl/ml) and stored at − 20 °C until RNA extraction.

### Primers design

**Fig. 1:**
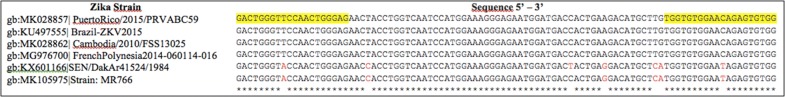
Alignment of the target amplicon of ZIKV NS5 gene. The nucleotides in red differ from the consensus and the asterisks indicate identity with the consensus sequence on the top. Nucleotides highlighted in yellow correspond to primer (NS5-2362F and NS5-2457R) annealing sites. Clustal Omega tool (EMBL-EBI - https://www.ebi.ac.uk/Tools/msa/clustalo/)

The ZIKV Nonstructural protein 5 (NS5), the polymerase used for viral RNA synthesis [61], was the selected target for primer design. An alignment of the ZIKV NS5 sequence from the Puerto Rico strain (GenBank: MK028857) was performed with ZIKV Brazil-2015 (GenBank: KU497555), Cambodia/2010 (GenBank: MK028862), FrenchPolynesia-2014 (GenBank: MG976700), Senegal-DakAr41524 (GenBank: KX601166) and MR766 (GenBank: MK105975) strains, using the Clustal Omega tool (European Bioinformatics Institute, EMBL-EBI: https://www.ebi.ac.uk/Tools/msa/clustalo/). In addition, an alignment of the ZIKV NS5 sequence (PRVABC59 strain, GenBank: MK028857) was performed with the *Ae. aegypti* and *Ae. albopictus* sequences from “EST”, “Assembled transcriptome” and “Transcripts” datasets of the VectorBase Bioinformatics Resource (https://www.vectorbase.org/). A highly conserved sequence region between analyzed ZIKV strains, but divergent from *Ae. aegypti* and *Ae. albopictus* mosquito genomes, was identified (Fig. 1). This sequence was applied as a template to design primers using the Primer-Blast tool (https://www.ncbi.nlm.nih.gov/tools/primer-blast/), the characteristics of the designed primers (NS5-2362F and NS5-2457R) are demonstrated on Table 1.

**Table 1:** Primers and probes used in the one-step RT-qPCR assay

Primer/probe	Gene product	Sequence (5′-3′)	Nucleotide position	Amplicon size (bp)	Reference
Zika virus					
NS5-2362F	NS5	GACTGGGTTCCAACTGGGAG	2362–2381	96	*
NS5-2457R	NS5	CCACACTCTGTTCCACACCA	2438–2457		
ZIKV 1086	Env	CCGCTGCCCAACACAAG	1086–1102	76	[15]
ZIKV 1162c	Env	CCACTAACGTTCTTTTGCAGACAT	1139–1162		
ZIKV 1107-FAM	Env	AGCCTACCTTGACAAGCAGTCAGACACTCAA	1107–1137		
ZIKV 1086-T7^1^	–	TAATACGACTCACTATAGGGAGACCGCTGCCCAACACAAG	–	–	*
NS5-2362-T7 F^1^	–	TAATACGACTCACTATAGGGAGAGACTGGGTTCCAACTGGGAG	–	–	*
*Aedes aegypti*					
DefA-F	Defensin A	AACTGCCGGAGGAAACCTAT	122–141	116	[62]
DefA-R	Defensin A	AATGCAATGAGCAGCACAAG	218–237		
ActF^2^	Actin	GAACACCCAGTCCTGCTGACA	583–603	65	[31]
ActR^2^	Actin	TGCGTCATCTTCTCACGGTTAG	626–647		
Act-FAM	Actin	AGGCCCCGCTCAACCCGAAG	605–624		

*Designed for the present study

^1^Primers containing T7 promoter sequence

^2^Primers used in both qRT-PCR methods (TaqMan and Sybr Green)

### One-step RT-qPCR assays

RNA was extracted from infected cells and/or *Ae. aegypti* using RNeasy Mini kit (Qiagen) according to manufacturer’s instructions, which included the use of the cell lysate biopolymer-shredding system (QIAshredder, Qiagen) and on-column deoxyribonuclease (DNase) digestion using RNase-free DNase I (Qiagen). RNA sample concentrations were measured using NanoDrop Spectrophotometer (Thermo Fisher Scientific). For ZIKV RNA detection, one-step RT-qPCR assays were performed on a CFX96 Touch Real-Time PCR Detection System (Bio-Rad, California, USA), using QuantiFast® SYBR® Green RT-PCR or QuantiFast® Probe RT-PCR kits (Qiagen) according to manufacturer’s instructions. Primers and probes used on RT-qPCR reactions are shown in Table 1. Forty nanograms of total RNA were used as template. The RT-qPCR cycling protocol consisted of an initial complementary deoxyribonucleic acid (cDNA) synthesis step at 50 °C for 10 min, a denaturation step at 95 °C for 5 min, followed by 50 replication cycles of 95 °C for 10 s and 60 °C for 30 s. When using QuantiFast® SYBR® Green RT-PCR kit, reactions were performed with primers at 400 nM instead of the concentration recommended by the manufacturer (1 µM). The absolute quantification of ZIKV RNA in mosquito samples was obtained using a standard curve constructed from *in vitro* transcribed RNA (as described below). *Aedes aegypti* Actin was used as control to confirm RNA integrity of samples submitted as well as for absolute quantification and normalization of infection results. Amplicon specificity of ZIKV NS5 was evaluated by the melting curve peak (80 ± 0.5 °C). The specificity of ZIKV NS5 primers was also evaluated by testing them on viral RNA of dengue virus (DENV-2 NGC strain) obtained from infected Vero cells and *Ae. aegypti* mosquitoes, as negative controls. The relative expression level of *Ae. aegypti* Defensin A was calculated by DeltaDelta CT (cycle threshold) method, using *Ae. aegypti* Actin as a gene reference. The primers utilized to amplify Defensin A are shown in Table 1.

### *In vitro* transcription of standard RNA

Five µg of RNA from Vero cells infected with ZIKV MR766 or PRVABC59 strain were used for synthesis of cDNA using SuperScript III Ribonuclease (RNase) H-Reverse Transcriptase (Invitrogen, California, USA). First strand cDNA was subjected to PCR using DreamTaq deoxyribonucleic acid (DNA) Polymerase (Thermo Fisher Scientific) with ZIKV 1086-T7/ZIKV 1162c or NS5-2362-T7 F/NS5-2457R primer sets (Table 1). The PCR products, containing T7 promoter sequence on the positive-sense strand, were used as DNA template for *in vitro* transcription using the Megascript kit (Ambion, Thermo Fisher Scientific), according to manufacturers’ instructions. The number of single strand RNA copies (molecules/μl) was calculated as described by Faye et al. [18].

## Results

### Identification of conserved ZIKV region divergent from *Aedes*

Aiming to design oligos optimized to minimize non-specific amplification in one-step qRT-PCR assay that allows the analysis of mosquito gene expression during ZIKV infection in the same samples, the ZIKV NS5 gene sequence of the PRVABC59 strain was submitted for sequence alignment analysis with *Ae. aegypti* and *Ae. albopictus* sequences from “EST”, “Assembled transcriptome” and “Transcripts” datasets from VectorBase, using the BLAST tool (https://www.vectorbase.org/blast). At that point, PRVABC59 NS5 sequences without similarity to mosquito genomic sequences were aligned with three ZIKV strains from the Asian phylogenetic lineage (Brazil, French Polynesia and Cambodia) and 2 ZIKV strains from African phylogenetic lineage (Uganda-MR766 and Senegal) using ClustalW. At the conclusion of the analysis, a region of 96 nucleotides from ZIKV NS5, highly conserved between ZIKV strains but divergent from *Ae. aegypti* and *Ae. albopictus*, was identified and utilized as template to design qRT-PCR primers (Fig. 1).

### Confirmation of ZIKV detection in mammalian cells

**Fig. 2:**
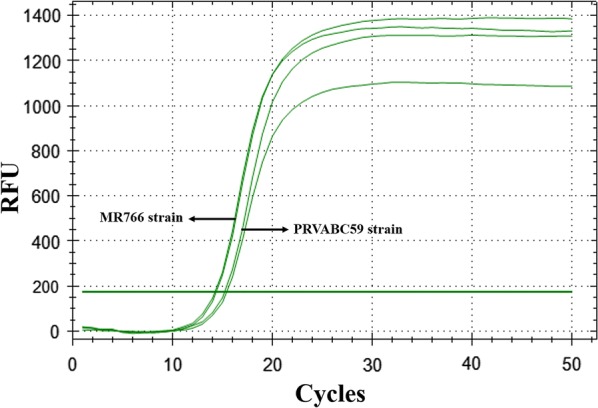
ZIKV strains detected from infected Vero cells. Total RNA from Vero cells infected with ZIKV MR766 and Puerto Rico strains was used as template for one-step RT-qPCR reaction, using NS5-2362F and NS5-2457R primers. Each sample was tested in duplicate

We performed qRT-PCR analysis using the designed NS5-2362F and NS5-2457R primers with RNA template isolated from Vero cells infected with ZIKV MR766 and PRVABC59 strains. In Fig. 2, the amplification plots for reactions using RNA from cells infected with both strains are shown, demonstrating that the NS5 primers can amplify ZIKV strains from Asian and African phylogenetic lineages.

### Assay sensitivity, reproducibility and specificity evaluation

The ZIKV 1086/ZIKV 1162c primers and the 1107-FAM probe [15] can be considered the gold standard to ZIKV RNA detection in mosquito samples since it has been preferentially used in most recent studies [31, 33–36, 38, 46, 49, 51]. The ZIKV 1086 and ZIKV 1162c have been successfully used to detect ZIKV RNA in cell lines, using SYBR Green as a dye instead the 1107-FAM [63] and could be a less expensive alternative to ZIKV detection in mosquitoes.

However, we observed nonspecific amplification in half of reactions using uninfected mosquito RNA as template, when using those primers in reaction with SYBR Green, while the reactions with NS5 primers did not present nonspecific detection (Table 2).

**Table 2:** Primers specificity evaluation in mosquito samples

Sample	PCR features	1107-FAM probe	ZIKV 1086 ZIKV 1162c primers	NS5 primers
Uninfected mosquitoes	Positive/tested	0/4	2/4	0/4
	Quantification cycle (Cq) value	–	30–31	–
	Melting temperature	–	78 °C	–
Infected mosquitoes	Positive/tested	4/4	4/4	4/4
	Quantification cycle (Cq) value	22–23	20–21	19–20
	Melting temperature	–	79 °C	79.5–80 °C
Positive control	Positive/tested	4/4	4/4	4/4
	Quantification cycle (Cq) value	20	17	18–19
	Melting temperature	–	79 °C	80 °C

*Notes*: Total RNA from control and ZIKV-infected (PRVABC59 strain) whole mosquitoes were extracted 7 days post-infection and used as template for qRT-PCR reactions, using 1107-FAM probe and NS5-2362F/NS5-2457R or ZIKV 1086/ZIKV 1162c primers. Positive controls consisted of total RNA from Vero cells infected with ZIKV PRVABC59 strain

**Fig. 3:**
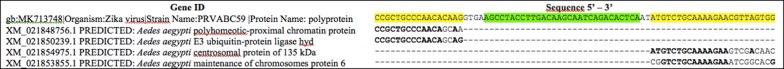
Alignment of the target amplicon amplified by ZIKV 1086 and ZIK 1162c primers with *Aedes aegypti* mRNA sequences. The nucleotides in bold on *Ae. aegypti* sequences indicate identities with consensus sequence on the top (Zika virus - PRVABC59 strain: polyprotein). Nucleotides highlighted in yellow correspond to primer (ZIKV 1086 and ZIK 1162c) annealing sites. Sequence highlighted in green correspond to 1107-FAM probe binding site

Once ZIKV 1086/ZIKV 1162c primers were designed to detect ZIKV RNA in human samples, the observed nonspecific amplifications could be caused by similarities between mosquito and viral sequences. Then, we performed the alignment of the sequence amplified by ZIKV 1086/ZIKV 1162c primers and *Ae. aegypti* sequences, using the BLAST tool (https://blast.ncbi.nlm.nih.gov/Blast.cgi). The identities found in four of many significant alignments obtained between the primers annealing sites and mRNA sequences of *Ae. aegypti* are demonstrated in Fig. 3. The results shown in Table 2 and Fig. 3 demonstrate the importance of optimization with primers design to detect ZIKV RNA in mosquito samples.

**Fig. 4:**
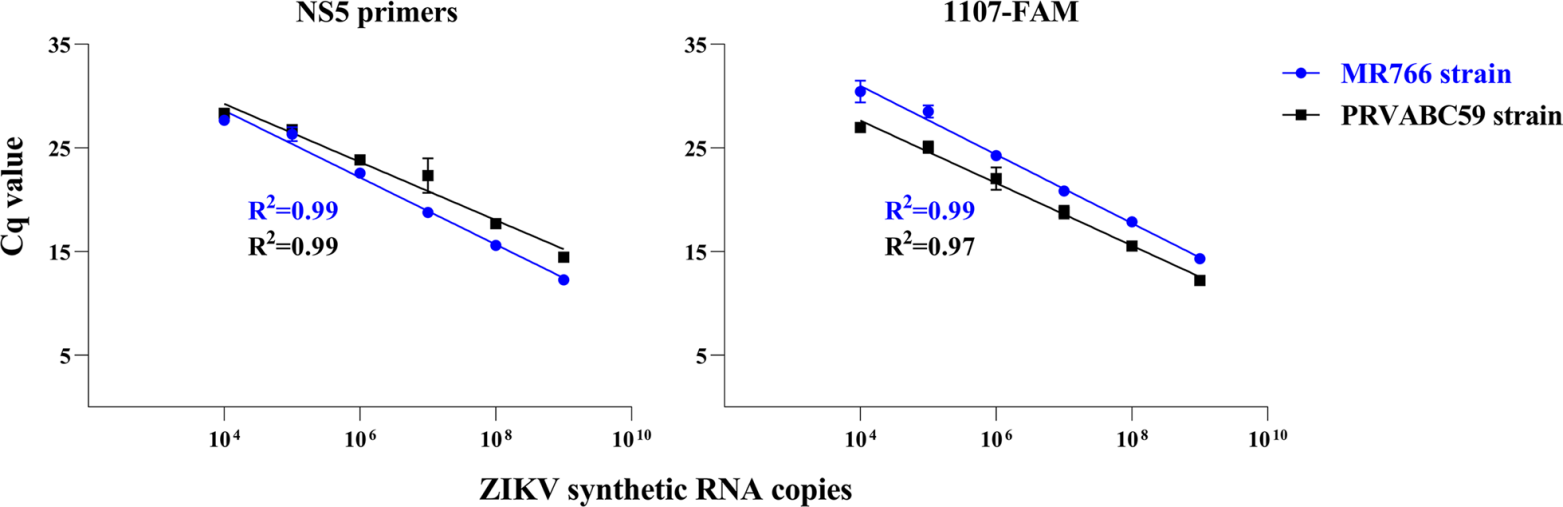
Limit of detection and efficiency of the one-step RT-qPCR assay for ZIKV RNA detection. RNAs transcribed *in vitro*, containing sequences of NS5 and Env genes from MR766 and PRVABC59 strains, were used as templates for RT-qPCR reactions using hydrolysis probe (1107-FAM) and SYBR Green (NS5-2362F and NS5-2457R primers) as fluorescent dyes. Cq values (mean ± standard deviation) were obtained from two technical replicates, performed in triplicate each. Coefficient of determination (*R*^2^) was calculated using GraphPad Prism 8.3.0 software

Positive single-strand RNAs, transcribed *in vitro*, were used to create standard curves to assess the limit of detection, efficiency and reproducibility of the RT-qPCR assay using NS5-2362F and NS5-2457R primers and SYBR Green as fluorescent dye. The gold standard used in the literature (ZIKV 1086/ZIKV 1162c primers and 1107-FAM as the dye) was used in comparisons; referred to in the paper as “1107-FAM probe”. The detection of the RT-qPCR reactions was linear over six 10-fold dilutions (10^9^ to 10^4^ copies/reaction) using synthetic RNA with sequences from the MR766 and PRVABC59 strains (Fig. 4).

The coefficient of determination (*R*^2^) from synthetic RNA standard curves was calculated to assess the efficiency of reactions using NS5-2362F and NS5-2457R primers, compared to the gold standard (1107-FAM probe). The results show that reactions using the NS5 primers are highly efficient (*R*^2^ = 0.99) in the detection of RNA sequences from both ZIKV strains tested (MR766 and PRVABC59) (Fig. 4). The determination coefficients obtained in reactions using 1107-FAM probe were 0.99 and 0.97 to MR766 and PRVABC59 strains, respectively.

**Fig. 5:**
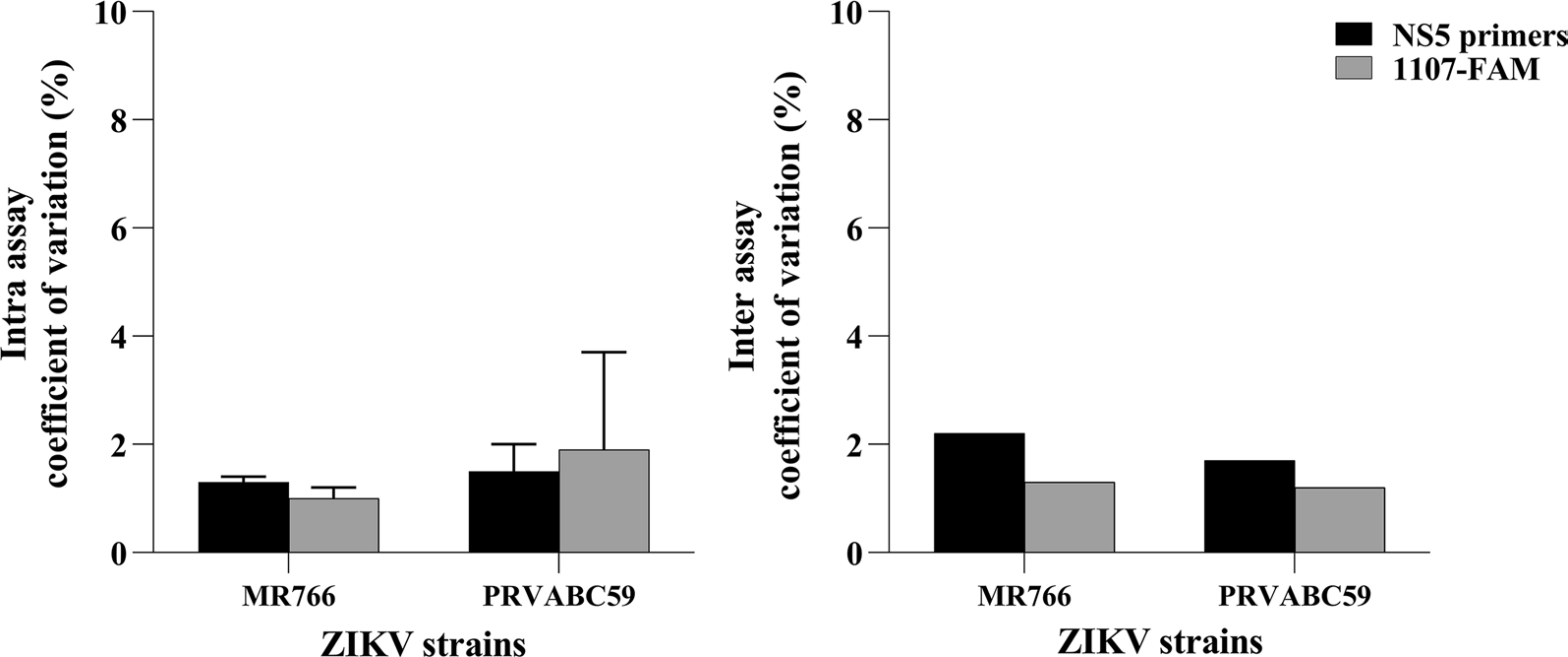
Reproducibility intra and inter assay of the one-step RT-qPCR assay for ZIKV RNA detection. RNAs transcribed *in vitro* (10^8^ copies/reaction), containing sequences of NS5 and Env genes from MR766 and PRVABC59 strains, were used as templates for RT-qPCR reactions using hydrolysis probe (1107-FAM) and SYBR Green (NS5-2362F and NS5-2457R primers) as fluorescent dyes. Coefficients of variance were calculated from three technical replicates, performed in triplicate each. Coefficient of variance = standard deviation/mean × 100

To further assess the efficiency and reproducibility of this assay, intra and inter assay coefficients of variation (CV) were calculated from quantification cycle (Cq) values obtained using 10^8^ copies/reaction of *in vitro* transcribed RNA, from ZIKV MR766 and PRVABC59 strains. These data are shown in Fig. 5. The intra assay coefficients of variation for reactions using NS5 primers were 1.3 ± 0.1% (MR766 strain) and 1.5 ± 0.5% (PRVABC59 strain) while the values obtained using 1107-FAM probe were 1.0 ± 0.2% (MR766 strain) and 1.9 ± 1.8% (PRVABC59 strain). The inter assay variation for reactions using NS5 primers were 2.2 and 1.7% for sequences from MR766 and PRVABC59 strains, respectively. When using 1107-FAM probe, the inter assay variations were 1.3% for MR766 strain and 1.2% for PRVABC59 strain.

The data shown in Figs. 4 and 5 demonstrate that the developed assay is highly reproducible and efficient, and this assay has the same level of sensitivity as the gold standard used in literature (1107-FAM probe).

### Assay detects same levels of ZIKV infection than the gold standard in mosquito samples

**Fig. 6:**
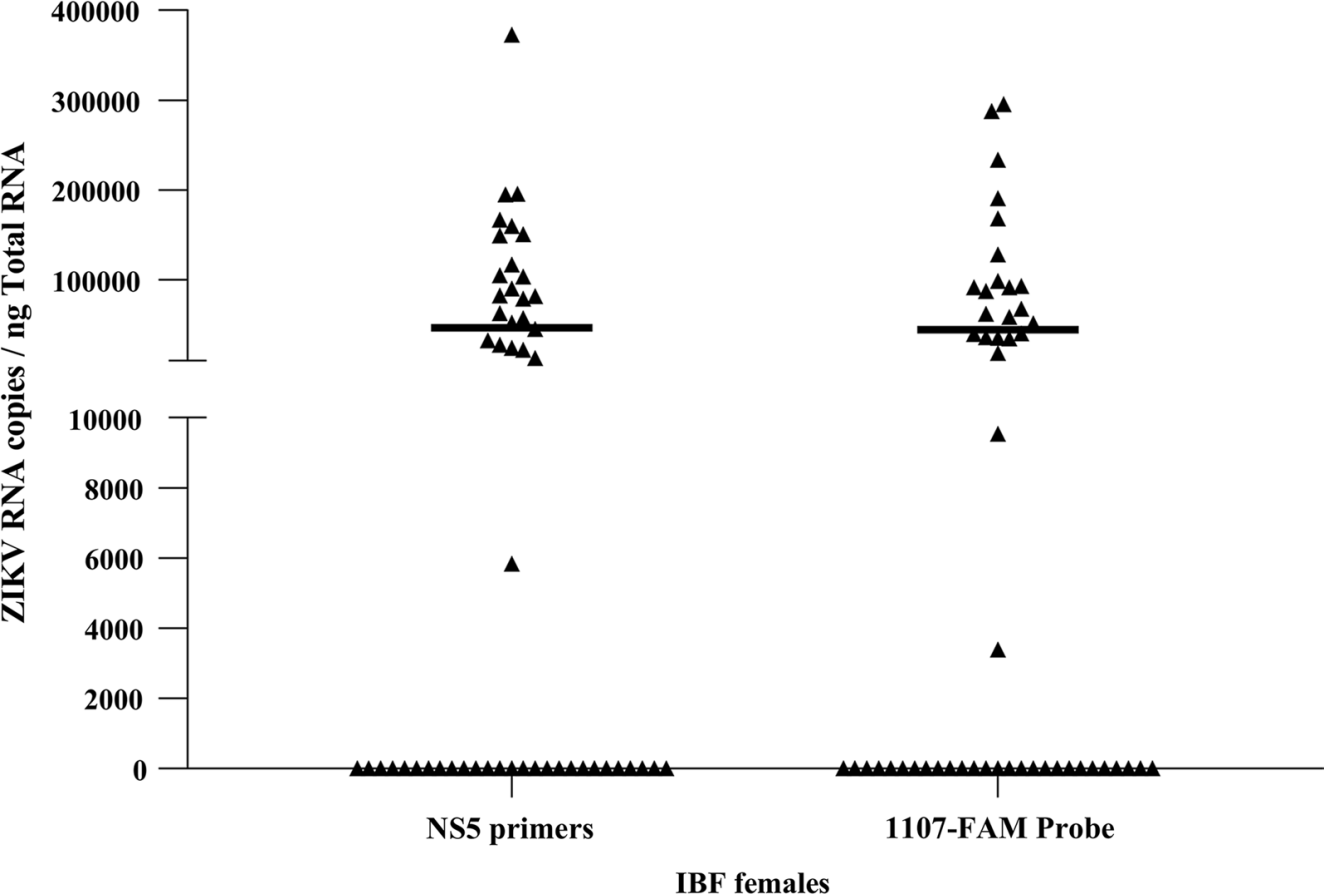
Detection of ZIKV from infected blood-fed (IBF) mosquitoes. Total RNA from ZIKV-infected (PRVABC59 strain) whole mosquitoes were extracted 7 days post-infection, and used as template for RT-qPCR reactions, using NS5-2362F/NS5-2457R primers and 1107-FAM probe. The absolute quantification of ZIKV RNA in mosquito samples was obtained using a standard curve constructed from *in vitro* transcribed RNA

The NS5-2362F and NS5-2457R primers were also tested in RT-qPCR reactions using RNA template isolated from infected blood-fed (IBF) female *Aedes* mosquitoes. Total RNA from ZIKV-infected whole mosquitoes (PRVABC59 strain) was extracted 7 days post-blood-feeding. RT-qPCR using the NS5-2362F and NS5-2457R primers showed an infection rate of approximately 47% (24 of 51 females), with infection levels of 9.9 × 10^4^ ± 8.1 × 10^4^ ZIKV RNA copies/ng total RNA. The mosquito samples were also analyzed using the 1107-FAM (gold standard) and the results demonstrate a highly approximate ZIKV RNA detection level (9.6 × 10^4^ ± 8.4 × 10^4^ copies/ng total RNA), as well as the same infection rate, with that obtained using NS5 primers (Fig. 6).

### Defensin A upregulation detected in orally infected *Ae. aegypti*

**Fig. 7:**
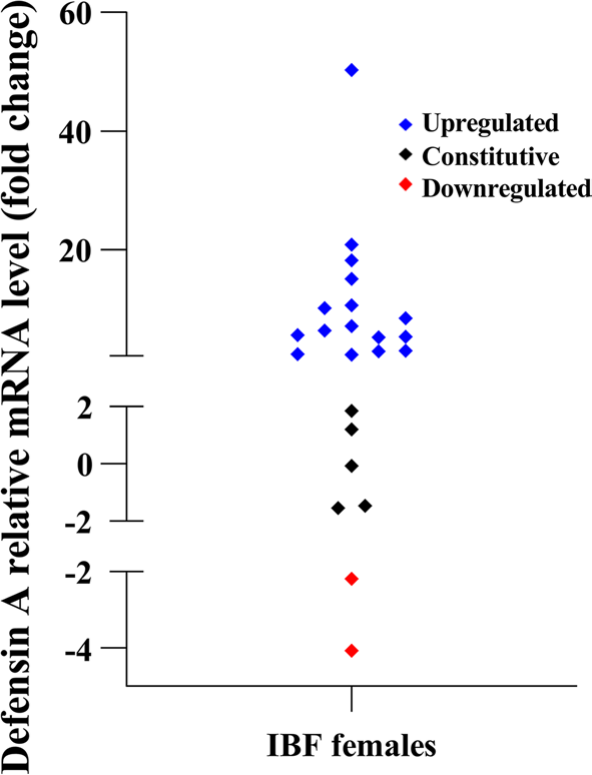
Defensin A gene expression in infected blood-fed (IBF) mosquitoes. Samples positive for ZIKV were used for RT-qPCR to evaluate Defensin A transcription levels. Actin was utilized as reference gene. Each sample was tested in duplicate

Since the *Ae. aegypti* antimicrobial peptide Defensin A has a role in the mosquito immune response to dengue, Chikungunya virus and Zika virus infection [64, 65], we chose to measure expression of this gene in our infected mosquitoes as confirmation that our assay allows for both ZIKV infection detection and analysis of gene expression in the same sample. RNA samples which tested positive for ZIKV were used in additional qRT-PCR analysis to evaluate the transcription of the gene Defensin A (data shown in Fig. 7). The results showed that from the ZIKV-positive mosquitoes Defensin A was upregulated in 69.6% (16 females), downregulated in 8.7% (2 females) and constitutive in 21.7% (5 females). In samples that displayed upregulation, the relative expression for Defensin A ranged from 2.2 to 50.3 in fold-change, with a mean of 10.3 ± 11.9 fold-change, corroborating results of Zhao et al. [64] that demonstrate Defensin A upregulation level of approximately 5 fold-change in pooled samples (10 females each) of ZIKV infected females.

## Discussion

With increasing outbreaks of ZIKV in humans globally and subsequent increase in experimental analysis of viral pathogenesis and interactions in the mosquito vector, an effective and reliable method to detect ZIKV infection in mosquito samples is essential. This study has presented a sensitive, efficient and reproducible assay for the detection of ZIKV RNA in *Ae. aegypti*, using one-step qRT-PCR and oligos based on the viral NS5 gene sequence. This assay also uniquely allows for the analysis of transcriptional levels of mosquito genes during ZIKV infection from the same samples. The method of ZIKV RNA detection in mosquitoes presented here is based on RT-qPCR using a standard RNA extraction kit, primers designed to avoid nonspecific amplification of the mosquito genome and SYBR green as a fluorescent label. Our protocol is relevant since the gold standard for the detection of ZIKV by qRT-PCR in the literature is based on viral RNA extraction kits and the use of hydrolysis probes as the fluorescent label, which is relatively effective but expensive, and the published primer and probe sets were optimized in assays using mammalian samples.

In recent studies that used qRT-PCR analysis to detect ZIKV RNA in mosquitoes, most used hydrolysis probes as fluorescent labels. More than half of the studies that used fluorescent probes to detect ZIKV RNA also used viral extraction kits and four did not describe how RNA extraction was performed [9, 14, 16, 18, 22, 23, 31–42, 44–46, 48, 49].

To date, only five studies [19, 21, 24, 43, 47] have detected ZIKV RNA in mosquitoes using SYBR green. However, in three of these studies [24, 43, 47] commercial kits designed to obtain mostly viral RNA from samples were used, which will not allow for analysis of mosquito gene expression from the same sample. In the one study that used a standard extraction kit along with SYBR green, did not present any analysis about primers specificity, sensitivity, efficiency or reproducibility [19].

In summary, from recent studies in which qRT-PCR was used to detect ZIKV RNA in mosquitoes, 83% of studies used hydrolysis probes as fluorescent labels and 56% describe use of viral RNA extraction kits (considering studies that do not specify extraction RNA method it can reach 70%). Although this approach has been demonstrated to be effective, it is also expensive. In addition, since the isolation of viral RNA is prioritized, this method does not permit the evaluation of the expression of mosquito genes during ZIKV infection. Studies that analyze ZIKV infection in mosquitoes, for example vector competence analyses, often require the assessment of different tissues at different time-points from individual mosquitoes. This work generates large numbers of samples, each requiring RNA extraction as well as multiple qRT-PCR reactions for sufficient analysis, and all of which using current methods come at great expense. Due to this financial barrier, the method presented in this study is an especially relevant alternative for the inexpensive, rapid and reliable detection of ZIKV in mosquito samples by qRT-PCR.

Considering that majority of ZIKV outbreaks occur in developing countries, the availability of highly sensitive, but also affordable, assays to detect ZIKV can greatly enhance local vector surveillance and disease control. Taking into account the experimental conditions and reagents utilized in this work, the prices of PCR reactions using NS5 primers were 31% less expensive than using 1107-FAM probe (USD 2.35/sample and USD 3.39/sample, respectively).

## Conclusions

The experimental approach developed and demonstrated in this study can significantly contribute to research efforts in study of mosquito gene expression during ZIKV infection. The assay used to detect ZIKV RNA in *Ae. aegypti* presented here has been demonstrated to be sensitive, efficient and reproducible for *in vitro* analysis in the laboratory as well as on infected mosquito samples. In addition, our presented protocol allows for the analysis of mosquito gene expression during ZIKV infection in the same samples and is a much less expensive approach optimized to minimize nonspecific amplification in *Aedes* spp. samples.

## Data Availability

All relevant data and material are available upon request.

## Abbreviations

ZIKV: Zika virus
qRT-PCR: Quantitative reverse transcription polymerase chain reaction
RNA: Ribonucleic acid
AMP: Antimicrobial peptide
DMEM: Dulbecco’s modified Eagle’s medium
ACL3: Arthropod containment level 3
PFU: Plaque-forming unit
NS5: Nonstructural protein 5
EMBL-EBI: European Bioinformatics Institute
DNase: Deoxyribonuclease
DENV: Dengue virus
DNA: Deoxyribonucleic acid
cDNA: Complementary deoxyribonucleic acid
CT: Threshold cycle
RNase: Ribonuclease
CV: Coefficient of variation
Cq: Quantification cycle
IBF: Infected blood-fed
